# Supplementary material for: An ATP-Binding Cassette Transporter, LaABCB11, Contributes to Alkaloid Transport in Lycoris aurea
Source: Int J Mol Sci. 2021 Oct 24;22(21):11458. doi: 10.3390/ijms222111458 (PMC8584037; doi:10.3390/ijms222111458)
Supplement: Supplementary file 1 [file ijms-22-11458-s001.zip › Supplemental information 1.pdf]

Supplemental information 1

>LaABCB11

ATGGGTATTGAAGAAGAGAAGGACAAAATTAGTCCTTCAGTCAATGGATCAAATGAGCCA
TCTTCTAGTAATTCAACGGTGAAAGAAGCAAACGGCGAGAAGAGCAATGAGAAAAGGGA
CGAGGCGAAGTACACCGTTCCGTTCTACAAGCTGTTCGCTTTTGCAGACTCGACAGATGTG
CTTCTCATGACCGCAGGCTCGATTGGAGCTGTGGCTAATGGGCTTGCATTGCCTCTCATGA
CGGTGCTCTTTGGAAATTTGATCCAATCTTTCGGAGGAGCTACTAATATTCATGATGTGGT
TCATAGGGTTTCCAAGGTCTGTCTTGAGTTTGTCTACTTGGCTGTGGGATCAGGGATAGCG
TCATTTTTTCAGGTGTCTTGTTGGATGGCGACGGGGGAGAGGCAGGCTGCAAGAATAAGG
AATTTGTATTTGAAAACCATATTACGGCAAGAAATCGCATTCTTTGACAAGGAAACAAAT
ACTGGAGAGGTTGTGGGGAGAATGTCAGGTGATACTGTTCTCATTCAAGATGCCATGGGT
GAAAAGGTTGGCAAGTTCATCCAGCTAGTATCAACATTCTTTGGGGGTTTTGTAGTTGCAT
TTGTTCAAGGATGGCTTCTCACTCTTGTGATGCTAACCACAATCCCGCTCCTCGTGATAGC
AGGCGCGGCAATGGGAGCCGCAATATCAAAAATGGCATCGATGGGGCAAACAGCTTATG
CGGAGGCAGCTGTTGTCGTTGAGCAAACAATCGGTTCAATTAGAACAGTTGCATCTTTTAC
TGGCGAAAAGCAATCGGTCATAAAGTACAAGAAATCTCTCAAGAGTGCTTATGAGTCTAG
TGTTCGAGAGAGTCTCGCTTCAGGATTAGGTCTTGGTACTGTAATGCTAATCATGTTTTGC
GGCTACGGGTTGGGAATTTGGTATGGGTCAAAATTGATATTGGATAAAGGCTACACTGGT
GCTGATGTAATCAACGTGATATTTGCAGTCTTGACAGGCTCATTTTCCTTGGGACAGGCAT
CTCCATGCTTGACGGCATTTGCAGCAGGGCAAGCCGCGGCATTCAAGATGTTTGAAACGA
TCAACAGAAAGCCAGAGATAGATGCAACCGATCCAACAGGGAAAAAACTAGATGACATA
GTTGGAGATGTAGAATTTAAAGATGTTTACTTCAGCTATCCAACAAGACAAGATGAGCAA
ATATTTAGAGGATTTTCTCTATTCATACAGCATGGCACGACTGTAGCTTTGGTCGGAGAGA
GTGGAAGCGGGAAGTCTACAGTTATAAGTCTGATTGAGAGATTTTATGACGCACAAGCAG
GTGAAGTTCTTATAGATGGCATAAACCTTAAGGAATTTCAACTCAAATGGATCAGAGGTA
AAATTGGGCTAGTGAGTCAAGAGCCCGTACTCTTTGCATCAAGCATTAGAGATAACATAG
CCTATGGAAAGGAAGGGGCAACCACTGAAGAAATTAGAGCCGCAACTGAGCTTGCTAAT
GCTTCAAAGTTTATAGACAAGATGCCACAGGGACTTGACACCATGGTTGGTGAGCATGGG
ACCCAGCTATCCGGGGGCCAAAAACAAAGGATTGCGATTGCAAGAGCTATTCTGAAAGAC
CCACGAATTCTGCTTCTTGATGAAGCCACCAGTGCTTTGGATGCAGAATCTGAAAGGATA
GTGCAAGAGGCACTTGATAGGATAATGGCAAACCGTACGACTGTCATTGTTGCTCATCGC
TTGAGTACAGTGAGAAATGCAGATACTATTGCTGTTATACACAGAGGTTCAATTGTGGAA
AAAGGTTCGCATTCCGAGCTACTAAAGGATTCAAATGGTGCTTATTGCCAACTCATACGCT
TGCAAGAAATGAATCAAAATTCTGACCATCCATCTCAGTCCAGCAAGGCTAAGCTCAATC
TTTCATCTGACGCTGGGAGGCGCTCTAGCAGACATATGTCTCTAAATCGTTCAATAAGCCG
TGAATCATCTTCTATAGGCAACAGTAGTCGCTCTTTCACATTACCCTTAGGATTGCCCCCA
GGAATTGATCTTCAAGAGACCAAATCAGAGGGAGCAACAAACACTGAGGTTCCTCCAGCA
CAAGTAAAGGAAGTATCACTCCGACGCCTTGCCTACCTCAATAAACCTGAAATTCCAGTTC
TTATAATTGGTGTGATCTCTGCTGTTGTTAATGGGCTTGTGTTTCCCATATTTGGACTACTC
TTATCGAGCGTGATAAATACATTCTACCAACCACCTGATAAACTCAAAAAGGATTCAAAA
TTTTGGTCGTTGTTGTTTGCTATATTTGGTGTGATTTCTTTCCTAGCACTTCCCGCTAGAACT
TACTTTTTTGGAGTGGCCGGAGCCAAATTAATACAAAGGATAAGATCAATGACCTTCGAG
AAGGTGGTACATATGGAGGTTGCTTGGTTTGATGAACCTGAAAATTCTAGTGGAGCAATT
GGCGCAAGATTATCAGCTGATGCAGCGACGGTTCGGGGCCTCGTGGGTGATGCACTTGCT

TTAGTTGTTCAGAACATTACAACTTTAATTGCTGGTTTGGTAATTGCTTTTGTTGCAAACTG
GCAATTGTCTTTTATTATCTTGGCTCTGATACCACTCATAGGCATCAACGGATATATCCAA
GTGAAGTTCATGACTGGATTTAGTGCTGATGCAAAGATGATGTATGAGGAGGCTAGTCAG
GTTGCCAATGATGCCGTGGGAAGTATAAGAACCGTTGCTTCTTTCTCAGCTGAAGATAAA
GTGATGGAACTTTACAAGAAGAAATGTGAAGGACCTATGAGGACTGGAATTCGGCAAGG
ATTGATTAGCGGTATTGGATTTGGAGCCTCCTTCTTCTTGCTATTTTGTGTTTATGCAACCA
GTTTTTATGCGGGAGCTCGCCTTGTGGAGGATGGAAAGACTACATTTGGAAAAGTTTTTAA
GGTTTTCTTTGCTCTTTCTATGGCAGCTATTGGAATTTCTCAATCAAGCTCCCTAGCGCCAG
ACTCTAGCAAAGCCAAATCCGCTACAGCTTCTGTGTTTGCTGTTCTTGATCGTAAGTCAAA
GATTGATCCAAGCGATGAATCCGGGATGACACTAGAAAGATTAAAGGGAAACATTGAGTT
CCGGCATGTCAGTTTCAAGTATCCAACAAGGCCACATGTGCAGATTTTCCAAGACTTGTGC
TTGTCGATTCATGCTGGAAAGACTGTTGCATTGGTTGGCGAGAGCGGAAGTGGGAAATCA
ACTGCGATAGCATTGTTACAAAGATTTTATGATCCCGATTCCGGTCATATACTGATAGATG
GAATGGAGATTGAGAAATTTCAGGTGCGGTGGCTAAGGCAGCAAATGGGTCTGGTTAGTC
AAGAACCAGCTTTGTTTAATGACACGATTCGAGCCAACATTGCCTACGGGAAAGAAGGAG
ATGCCACCGAGGCTGAAGTTGTAGCTGCGGCCGAGTCAGCAAATGCCCACAAGTTCATAT
GCAGTTTACAACAGGGTTATGACACGGTGGTTGGAGAGCGAGGGATCCAGCTATCAGGTG
GTCAAAAGCAACGGGTGGCGATTGCACGTGCCATTGTGAAAGAGCCAAAAATTCTACTTC
TAGACGAAGCAACAAGCGCGCTCGATGCTGAATCTGAACGAGTGGTTCAAGATGCTTTAG
ATCGAGTCATGGTCAACCGTACAACAATAGTGATCGCACATCGGTTGTCTACGATCAGAG
GTGCCGATGTGATTGCAGTGGTTAAGAATGGAATGATTATTGAGAAAGGAAACCATGATT
CATTGATCAATATCAAGGATGGTGCTTATGCATCATTAGTTGCACTTCACACAAATTCTTC
TACGTAG

>ΔLaABCB11
ATGGGTATTGAAGAAGAGAAGGACAAAATTAGTCCTTCAGTCAATGGATCAAATGAGC
CATCTTCTAGTAATTCAACGGTGAAAGAAGCAAACGGCGAGAAGAGCAATGAGAAAA
GGGACGAGGCGAAGTACACCGTTCCGTTCTACAAGCTGTTCGCTTTTGCAGACTCGAC
AGATGTGCTTCTCATGACCGCAGGCTCGATTGGAGCTGTGGCTAATGGGCTTGCATTGC
CTCTCATGACGGTGCTCTTTGGAAATTTGATCCAATCTTTCGGAGGAGCTACTAATATTC
ATGATGTGGTTCATAGGGTTTCCAAGGTCTGTCTTGAGTTTGTCTACTTGGCTGTGGGA
TCAGGGATAGCGTCATTTTTTCAGGTGTCTTGTTGGATGGCGACGGGGGAGAGGCAGG
CTGCAAGAATAAGGAATTTGTATTTGAAAACCATATTACGGCAAGAAATCGCATTCTTT
GACAAGGAAACAAATACTGGAGAGGTTGTGGGGAGAATGTCAGGTGATACTGTTCTC
ATTCAAGATGCCATGGGTGAAAAGGTTGGCAAGTTCATCCAGCTAGTATCAACATTCTT
TGGGGGTTTTGTAGTTGCATTTGTTCAAGGATGGCTTCTCACTCTTGTGATGCTAACCA
CAATCCCGCTCCTCGTGATAGCAGGCGCGGCAATGGGAGCCGCAATATCAAAAATGGC
ATCGATGGGGCAAACAGCTTATGCGGAGGCAGCTGTTGTCGTTGAGCAAACAATCGGT
TCAATTAGAACAGTTGCATCTTTTACTGGCGAAAAGCAATCGGTCATAAAGTACAAGA
AATCTCTCAAGAGTGCTTATGAGTCTAGTGTTCGAGAGAGTCTCGCTTCAGGATTAGGT
CTTGGTACTGTAATGCTAATCATGTTTTGCGGCTACGGGTTGGGAATTTGGTATGGGTCA
AAATTGATATTGGATAAAGGCTACACTGGTGCTGATGTAATCAACGTGATATTTGCAGTC
TTGACAGGCTCATTTTCCTTGGGACAGGCATCTCCATGCTTGACGGCATTTGCAGCAGG
GCAAGCCGCGGCATTCAAGATGTTTGAAACGATCAACAGAAAGCCAGAGATAGATGC

AACCGATCCAACAGGGAAAAAACTAGATGACATAGTTGGAGATGTAGAATTTAAAGAT
GTTTACTTCAGCTATCCAACAAGACAAGATGAGCAAATATTTAGAGGATTTTCTCTATTC
ATACAGCATGGCACGACTGTAGCTTTGGTCACAGTTATAAGTCTGATTGAGAGATTTTAT
GACGCACAAGCAGGTGAAGTTCTTATAGATGGCATAAACCTTAAGGAATTTCAACTCA
AATGGATCAGAGGTAAAATTGGGCTAGTGAGTCAAGAGCCCGTACTCTTTGCATCAAG
CATTAGAGATAACATAGCCTATGGAAAGGAAGGGGCAACCACTGAAGAAATTAGAGCC
GCAACTGAGCTTGCTAATGCTTCAAAGTTTATAGACAAGATGCCACAGGGACTTGACA
CCATGGTTGGTGAGCATGGGACCCAGCTATCCGGGGGCCAAAAACAAAGGATTGCGAT
TGCAAGAGCTATTCTGAAAGACCCACGAGCCACCAGTGCTTTGGATGCAGAATCTGAA
AGGATAGTGCAAGAGGCACTTGATAGGATAATGGCAAACCGTACGACTGTCATTGTTG
CTCATCGCTTGAGTACAGTGAGAAATGCAGATACTATTGCTGTTATACACAGAGGTTCA
ATTGTGGAAAAAGGTTCGCATTCCGAGCTACTAAAGGATTCAAATGGTGCTTATTGCCA
ACTCATACGCTTGCAAGAAATGAATCAAAATTCTGACCATCCATCTCAGTCCAGCAAGG
CTAAGCTCAATCTTTCATCTGACGCTGGGAGGCGCTCTAGCAGACATATGTCTCTAAAT
CGTTCAATAAGCCGTGAATCATCTTCTATAGGCAACAGTAGTCGCTCTTTCACATTACCC
TTAGGATTGCCCCCAGGAATTGATCTTCAAGAGACCAAATCAGAGGGAGCAACAAACA
CTGAGGTTCCTCCAGCACAAGTAAAGGAAGTATCACTCCGACGCCTTGCCTACCTCAA
TAAACCTGAAATTCCAGTTCTTATAATTGGTGTGATCTCTGCTGTTGTTAATGGGCTTGT
GTTTCCCATATTTGGACTACTCTTATCGAGCGTGATAAATACATTCTACCAACCACCTGA
TAAACTCAAAAAGGATTCAAAATTTTGGTCGTTGTTGTTTGCTATATTTGGTGTGATTTC
TTTCCTAGCACTTCCCGCTAGAACTTACTTTTTTGGAGTGGCCGGAGCCAAATTAATAC
AAAGGATAAGATCAATGACCTTCGAGAAGGTGGTACATATGGAGGTTGCTTGGTTTGAT
GAACCTGAAAATTCTAGTGGAGCAATTGGCGCAAGATTATCAGCTGATGCAGCGACGG
TTCGGGGCCTCGTGGGTGATGCACTTGCTTTAGTTGTTCAGAACATTACAACTTTAATT
GCTGGTTTGGTAATTGCTTTTGTTGCAAACTGGCAATTGTCTTTTATTATCTTGGCTCTG
ATACCACTCATAGGCATCAACGGATATATCCAAGTGAAGTTCATGACTGGATTTAGTGCT
GATGCAAAGATGATGTATGAGGAGGCTAGTCAGGTTGCCAATGATGCCGTGGGAAGTA
TAAGAACCGTTGCTTCTTTCTCAGCTGAAGATAAAGTGATGGAACTTTACAAGAAGAA
ATGTGAAGGACCTATGAGGACTGGAATTCGGCAAGGATTGATTAGCGGTATTGGATTTG
GAGCCTCCTTCTTCTTGCTATTTTGTGTTTATGCAACCAGTTTTTATGCGGGAGCTCGCC
TTGTGGAGGATGGAAAGACTACATTTGGAAAAGTTTTTAAGGTTTTCTTTGCTCTTTCT
ATGGCAGCTATTGGAATTTCTCAATCAAGCTCCCTAGCGCCAGACTCTAGCAAAGCCAA
ATCCGCTACAGCTTCTGTGTTTGCTGTTCTTGATCGTAAGTCAAAGATTGATCCAAGCG
ATGAATCCGGGATGACACTAGAAAGATTAAAGGGAAACATTGAGTTCCGGCATGTCAG
TTTCAAGTATCCAACAAGGCCACATGTGCAGATTTTCCAAGACTTGTGCTTGTCGATTC
ATGCTGGAAAGACTGTTGCATTGGTTACTGCGATAGCATTGTTACAAAGATTTTATGATC
CCGATTCCGGTCATATACTGATAGATGGAATGGAGATTGAGAAATTTCAGGTGCGGTGG
CTAAGGCAGCAAATGGGTCTGGTTAGTCAAGAACCAGCTTTGTTTAATGACACGATTC
GAGCCAACATTGCCTACGGGAAAGAAGGAGATGCCACCGAGGCTGAAGTTGTAGCTG
CGGCCGAGTCAGCAAATGCCCACAAGTTCATATGCAGTTTACAACAGGGTTATGACAC
GGTGGTTGGAGAGCGAGGGATCCAGCTATCAGGTGGTCAAAAGCAACGGGTGGCGAT
TGCACGTGCCATTGTGAAAGAGCCAAAAGCAACAAGCGCGCTCGATGCTGAATCTGA
ACGAGTGGTTCAAGATGCTTTAGATCGAGTCATGGTCAACCGTACAACAATAGTGATCG
CACATCGGTTGTCTACGATCAGAGGTGCCGATGTGATTGCAGTGGTTAAGAATGGAATG

ATTATTGAGAAAGGAAACCATGATTCATTGATCAATATCAAGGATGGTGCTTATGCATCA
TTAGTTGCACTTCACACAAATTCTTCTACGTAG

> probe
GGTTATGACACGGTGGTTGGAGAGCGAGGGATCCAGCTATCAGGTGGTCAAAAGCAA
CGGGTGGCGATTGCACGTGCCATTGTGAAAGAGCCAAAAATTCTACTTCTAGACGAAG
CAACAAGCGCGCTCGATGCTGAATCTGAACGAGTGGTTCAAGATGCTTTAGATCGAGT
CATGGTCAACCGTACAACAATAGTGATCGCACATCGGTTGTCTACGATCAGAGGTGCCG
ATGTGATTGCAGTGGTT
