# Supplementary material for: An ATP-Binding Cassette Transporter, LaABCB11, Contributes to Alkaloid Transport in Lycoris aurea
Source: Int J Mol Sci. 2021 Oct 24;22(21):11458. doi: 10.3390/ijms222111458 (PMC8584037; doi:10.3390/ijms222111458)
Supplement: Supplementary file 1 [file ijms-22-11458-s001.zip › Table S1 Primer.pdf]

Table S1 Primer summarization

Primer	Sequence
ABCB11-s	ATGGGTATTGAAGAAGAGAAGG
ABCB11-a	CGTAGAAGAATTTGTGTGAA
RT-s	TATGACACGGTGGTTGGAGA
RT-a	CCACTCGTTCAGATTCAGCA
TIP41-s	GCAACCATCCAAAGTTTAACTGCT
TIP41-a	AATGTGCAAGCAGGGCTAGTAA
11-1s	ATGGGTATTGAAGAAGAGAA
11-1a	TCAATCAGACTTATAACTGTGACCAAAGCTACAGTCGTGC
11-2s	GCACGACTGTAGCTTTGGTCACAGTTATAAGTCTGATTGA
11-2a	GCATCCAAAGCACTGGTGGCTCGTGGGTCTTTCAGAATAG
11-3s	CTATTCTGAAAGACCCACGAGCCACCAGTGCTTTGGATGC
11-3a	TGTAACAATGCTATCGCAGTAACCAATGCAACAGTCTTTC
11-4s	GGAAAGACTGTTGCATTGGTTACTGCGATAGCATTGTTACA
11-4a	GCATCGAGCGCGCTTGTTGCTTTTGGCTCTTTCACAATGGC
11-5s	CCATTGTGAAAGAGCCAAAAGCAACAAGCGCGCTCGATGC
11-5a	CTACGTAGAAGAATTTGTGT
196-11-s	tataccccagcctcgactagtATGGGTATTGAAGAAGAGAAGGACA
196-11-a	gataagcttgatatcgaattcCTACGTAGAAGAATTTGTGTGAAGTGC
1301-11-s	taagtccggagctagctctagaATGGGTATTGAAGAAGAGAAGG

1301-11-a	cgcccttgctcaccatggatccGTAGAAGAATTTGTGTG
probe-s	GGTTATGACACGGTGGTTGG
probe-T7-a	GCGAAATTAATACGACTCACTATAGGGAGAAACCACTGCAATCACATCGG
probe-T7-s	GCGAAATTAATACGACTCACTATAGGGAGAGGTTATGACACGGTGGTTGG
probe-a	AACCACTGCAATCACATCGG
